# Waist-to-Height Ratio Cut-Off Points for Central Obesity in Individuals with Overweight Across Different Ethnic Groups in NHANES 2011–2018

**DOI:** 10.3390/nu16223838

**Published:** 2024-11-08

**Authors:** Leila Itani, Marwan El Ghoch

**Affiliations:** 1 Department of Nutrition and Dietetics, Faculty of Health Sciences, Beirut Arab University, Riad El Solh, Beirut P.O. Box 11-5020, Lebanon; l.itani@bau.edu.lb; 2 Center for the Study of Metabolism, Body Composition and Lifestyle, Department of Biomedical, Metabolic and Neural Sciences, University of Modena and Reggio Emilia, 41125 Modena, Italy

**Keywords:** BMI, body composition, obesity, body fat, visceral adipose tissue

## Abstract

Background: The identification of surrogate measures of central obesity is of clinical importance, and the waist-to-height ratio (WtHR) has recently attracted great interest as an alternative method. Objective: For this reason, we aimed to establish specific WtHR cut-off points for adiposity (i.e., central obesity) in four different ethnicity groups across both sexes based on data from the National Health and Nutrition Examination Survey (NHANES) population. Methods: Of the total 23,037 participants who completed four cycles of the survey between the years 2011 and 2018, anthropometric measures (i.e., body weight, waist circumference, and height) and dual X-ray absorptiometry-derived visceral adipose tissue (DXA-derived VAT) results were available for 3566 individuals who were assessed in this cross-sectional study. Participants with an overweight status defined according to the World Health Organization (WHO) body mass index (BMI) cut-off points (25–29.9 kg/m^2^) were included. The sample was then categorized by adiposity according to the DXA-derived VAT tertiles (highest), and based on the receiver operating characteristic (ROC) curve analysis, the best sensitivity and specificity were attained for predicting central obesity using the WtHR. Results: The following WtHR cut-offs were identified as having the best discriminating ability for central obesity: 0.57 for White males and 0.58 for White females; 0.55 for Black males and 0.57 for Black females; 0.56 for Asian males and 0.59 for Asian females; and 0.57 for Hispanic males and 0.59 for Hispanic females. Conclusions: These new WtHR cut-off points should be utilized in adults with overweight to screen for central adiposity based on their sex and ethnicity, and obesity guidelines therefore need to be revised accordingly.

## 1. Introduction

Obesity is a growing health problem, and its prevalence is continually increasing worldwide [1]. It is associated with several medical [2] and psychosocial diseases [3,4] that increase the risks of morbidity and mortality [5]. Hence, the early identification and treatment of obesity is vital and has clinical importance [6]. In fact, the international guidelines on the topic recommend a wide range of weight loss interventions such as lifestyle modification programs (i.e., behavioral and cognitive behavioral ones) [7], as well as several anti-obesity drugs (i.e., glucagon-like peptide-1 [GLP-1] and gastric inhibitory polypeptide agonists) [8] and bariatric surgery (sleeve gastrectomy or mini gastric bypass) [9].

Although obesity is best defined as an excessive and abnormal fat deposition in the adipose tissue [10,11], the World Health Organization (WHO) still relies on body mass index (BMI) to classify individuals’ adiposity based on universal cut-off points for all adults, regardless of their ethnicity, age, or gender [12]. Specifically, the WHO BMI classification system considers a unique cut-off point of 30 kg/m^2^ to be indicative of obesity in White, Hispanic, and Black populations across all age groups (i.e., young, middle, and older adults) and both genders (i.e., males and females) [12]. However, this traditional classification system has always been subject to criticism [13] due to several limitations [14,15], such as that it is not suitable for all ethnicities (e.g., Asians) [16] and its inability to discriminate between the body compartments (e.g., bone, fat, and muscle) [17]. The identification of obesity based on body fat (BF) quantity and distribution therefore remains the most accurate method [18]. For instance, the visceral adipose tissue (VAT) is a component of total body fat, with high secreting activity by a large spectrum of adipokines related to inflammatory processes [19,20]. A significant deposition of VAT is known as visceral obesity [21] and is strongly associated with several obesity-related complications [22,23,24]. For this reason, VAT has attracted clinical and research interest and is considered a direct expression of central obesity, for which some papers have tried to suggest relative cut-off points regardless of body mass index (BMI) levels and total BF [25,26]. However, an accurate measurement of VAT requires sophisticated techniques such as a computed tomography (CT) scan or magnetic resonance imaging (MRI), which are not always available in all clinical settings, especially those related to nutrition [27].

As a result, some surrogate indices based on anthropometric measurements have been proposed, such as the waist circumference (WC) [28], waist-to-height ratio (WtHR) [29], and abdominal bioimpedance [27], as well as equations for VAT estimation [30]. For instance, the European Association for the Study of Obesity (EASO) has recently recommended a new framework for the diagnosis, staging, and management of obesity in adults, suggesting a WtHR ≥ 0.5 in people with a BMI between 25 kg/m^2^ and 29.9 kg/m^2^ as an expression of high adiposity for use in all ethnic groups across both sexes [31]. However, this cut-off point (i.e., WtHR ≥ 0.5) is not widely accepted, as even if it may be suitable for certain populations, its validity is not certain in others, and in some it may be inaccurate and generic [32]. Testing its accuracy, especially across different ethnic groups, is therefore strongly demanded.

Based on these considerations, the current study therefore aims to establish suitable WtHR cut-off points across different ethnic groups using data derived from the National Health and Nutrition Examination Survey (NHANES) of the non-institutionalized US population. We hypothesize that the WtHR cut-off point ≥ 0.5 is probably low for people with overweight (BMI ≥ 25 but <30 kg/m^2^), and higher cut-offs should be recommended.

## 2. Materials and Methods

### 2.1. Participants and the Design of the Study

The National Health and Nutrition Examination Survey (NHANES) is a large ongoing dietary survey of a nationally representative sample of the non-institutionalized US population [33]. It is conducted by the Centers for Disease Control and Prevention (CDC) and National Center for Health Statistics (NCHS) and has been monitoring the nation’s nutrition and health for more than five decades by collecting data through interviews, standard exams, and biospecimen collection [33]. Detailed descriptions of the survey design and the data collection procedures have been reported extensively elsewhere [33]. The variables of interest in our study are reported below.

**Figure 1:**
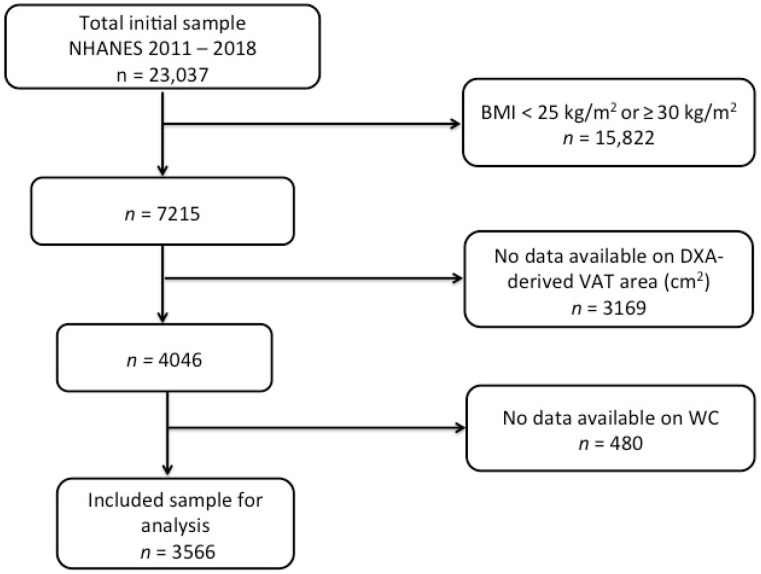
Flowchart and shift work analysis. Abbreviations: NHANES, National Health and Nutrition Examination Survey; BMI = body mass index; VAT = visceral adipose tissue; WC = waist circumference.

This was a cross-sectional study. Our final sample included 3566 participants from the National Health and Nutrition Examination Survey (NHANES, *n* = 23,037) who were non-institutionalized civilian adults below the age of 60 years residing in the United States across four different survey cycles between the years 2011 and 2018. The inclusion criteria were the following: individuals (i) with an age ≥ 20 years, (ii) who are within the WHO BMI classification for overweight (BMI ≥ 25 kg/m^2^), (iii) and have completed anthropometric measurements (i.e., body weight, waist circumference, and height) and (iv) body composition assessment by means of a dual X-ray absorptiometry (DXA) scan (i.e., DXA-derived VAT) (Figure 1). The exclusion criteria included having (i) an age < 20 years, (ii) an underweight or normal weight (<25 kg/m^2^) or obesity status (BMI ≥ 30 kg/m^2^) according to the WHO, and (iii) missing and unavailable WC, DXA-derived VAT, or data (Figure 1).

### 2.2. Data Collection

#### 2.2.1. Anthropometric and Demographic Data and Body Composition Assessment

Trained health technicians measured the participants’ waist circumference (WC), weight, and height. The WtHR was calculated as the WC in cm to height in cm and BMI as weight in kg to the square of height in m. The DXA-derived VAT area was expressed as cm^2^ between the fourth and fifth lumbar vertebrae, as defined by the Hologic APEX 4.0 software used in DXA scan analysis. Age was reported in years, sex as male or female, and ethnicity characteristics as White, Black, Hispanic, or Asian.

#### 2.2.2. Statistical Analysis

The statistical analysis was performed based on the analytic guidelines of the Centers for Disease Control [34]. Descriptive statistics are presented as means and standard deviations for continuous variables and frequencies and proportions for categorical ones. The association between the WtHR and DXA-derived VAT area (cm^2^) was tested with Pearson’s correlation coefficient. A positive linearity association between the WtHR and DXA-derived VAT area was confirmed by the cumulative sum (CUSUM) linearity test with a *p*-value > 0.05 [35]. A classification analysis, using the receiver operating characteristic (ROC) curve, was performed to evaluate the diagnostic performance of the WtHR in detecting central obesity. The sensitivity, specificity, and area under the curve (AUC) were calculated for the ROC curve. Central obesity was defined as the DXA-derived VAT area falling within the age-, sex-, and ethnicity-specific third tertile of the DXA-derived VAT area utilized as a gold standard [29]. The criterion values of the WtHR with maximum sensitivity and specificity as well as the shortest distance from the corner of the ROC curve were selected for the sex- and ethnicity-specific WtHR cut-off points. An AUC > 0.8 indicated that the criterion value has an excellent discriminating ability, and an AUC between 0.7 and 0.8 denoted an acceptable one [36]. All the tests were considered significantly different at *p* < 0.05.

To compare the performance of the new defined cut-off points and the arbitrarily proposed cut-off in the literature (0.5), the difference between the true positive rates was determined between the two cut-off points. The Number Cruncher Statistical System (NCSS) 12.02 (NCSS, Kaysville, UT, USA) package was employed for the statistical analysis [37]. A post hoc power analysis for the sample size was conducted with PASS 11 software [38]. For each of the subsamples from different ethnicities and sexes, given the number falling within the highest DXA-derived VAT tertile (TP) versus those in lower ones (TN) with an alpha of 0.05 and the observed AUC, the power was 1.000.

## 3. Results

The study sample included 2077 male (58.2%) and 1489 female (41.8%) individuals with a mean age of 40.6 ± 11.1 years and 40.7 ± 11.2 years, respectively. The mean BMI was 27.4 ± 1.4 kg/m^2^ in males and females and did not vary across ethnicities (Table 1, Table 2 and Table 3).

The mean DXA-derived VAT area was 105.4 ± 39.3 cm^2^ among males and 93.6 ± 37.0 cm^2^ among females. The WtHR was lower among males (0.56 ± 0.04) compared to females (0.58 ± 0.04). Over 90% of both males and females across almost all ethnicities, as well as over 75% of Black males, were classified as above the arbitrary WtHR (0.5) cut-off point proposed by the literature (Table 2 and Table 3).

**Figure 2:**
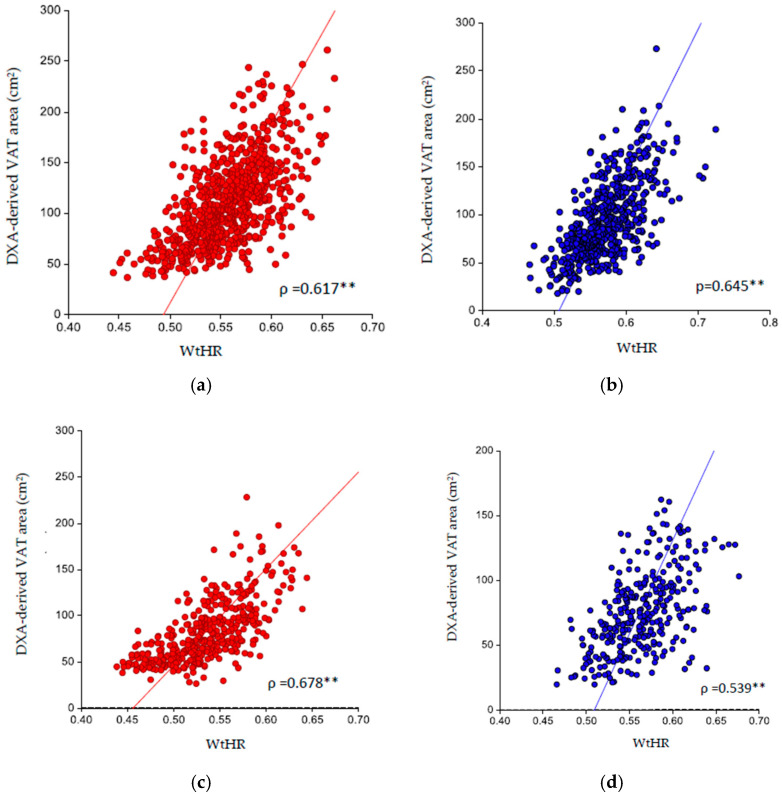

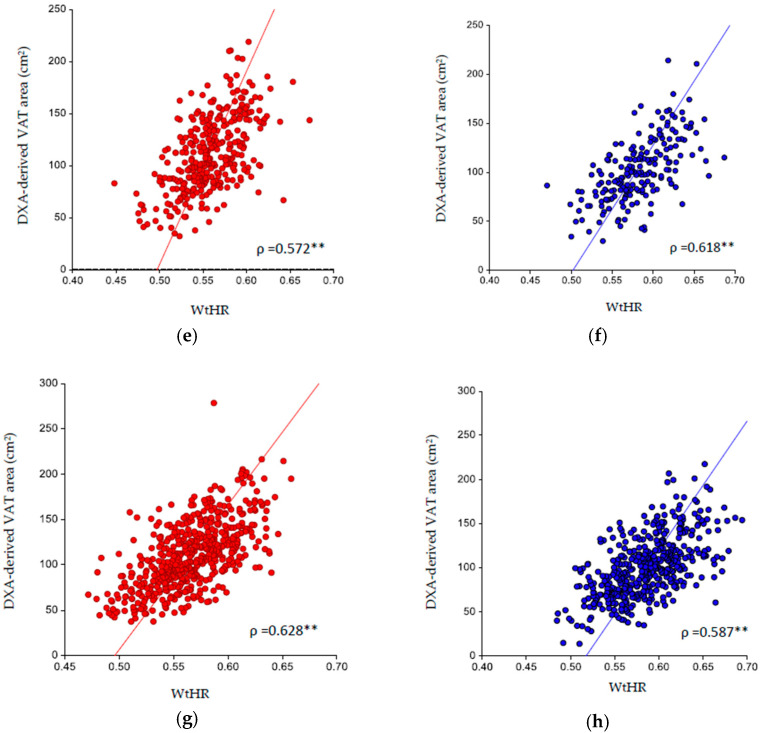
Association between DXA-derived VAT area (cm^2^) and WtHR in (**a**) White males; (**b**) White females; (**c**) Black males; (**d**) Black females; (**e**) Asian males; (**f**) Asian females; (**g**) Hispanic males; and (**h**) Hispanic females. WtHR = waist-to-height ratio; DXA-derived VAT area (cm^2^). ** *p*-value > 0.01.

The correlation analysis revealed a significant positive correlation between the WtHR and the DXA-derived VAT area among males and females from different ethnicities (Figure 2a–g). Furthermore, a significant positive linear association (Figure 2a–g) between the DXA-derived VAT area and WtHR in different ethnicities was confirmed by the CUSUM linearity test (*p* > 0.05).

**Figure 3:**
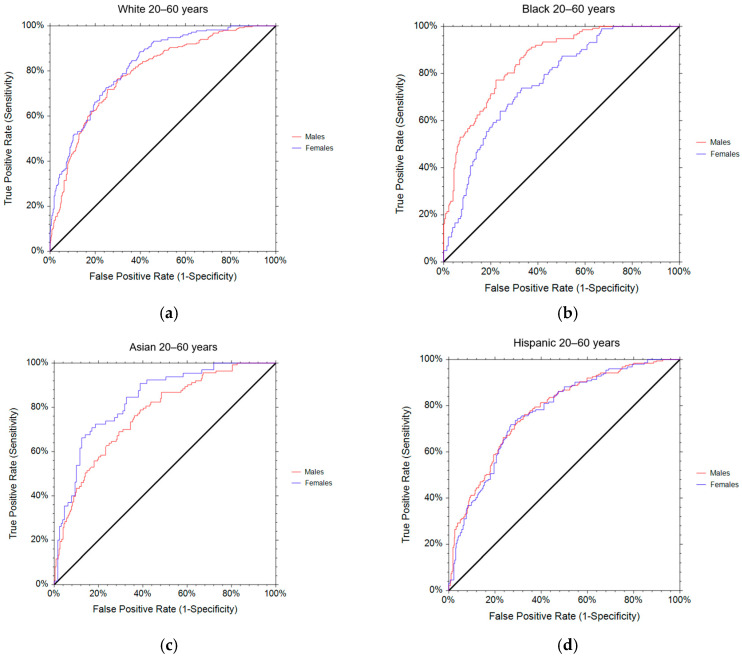
Receiver operator characteristics curve by sex and ethnicity for WtHR to detect central obesity (DXA-derived VAT area): (**a**) White; (**b**) Black; (**c**) Asian; and (**d**) Hispanic.

**Figure 4:**
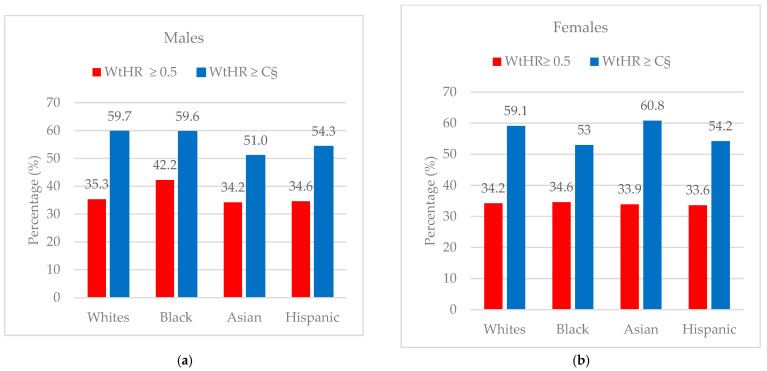
Distribution of correctly diagnosed (true positive) cases by the new ethnicity and sex specific cut-off point by (**a**) males and (**b**) females. WtHR = waist-to-height ratio; C^§^ = new sex- and ethnicity-specific WtHR cut-off point.

The results of the ROC analysis for the diagnostic performance of the WtHR by sex and ethnicity are presented in Table 4 and Figure 3a–d. Among White males, the mean age was 40.8 ± 11.1 years, the mean DXA-derived VAT area was 111.2 ± 41.8 cm^2^, and the mean WtHR was 0.56 ± 0.04. The majority (93.8%) were classified as having central obesity based on the arbitrary cut-off point (≥0.5) (Table 2). The ROC analysis for White males showed that the most appropriate cut-off for identifying central obesity based on the DXA-derived VAT area tertiles was 0.57 (Table 4). This cut-off point achieved good sensitivity (71.8%) and specificity (74.6%). Comparatively, the proportion correctly diagnosed by the new cut-off point was improved by 24.4%, reaching almost 60% (59.7%) (Table 5 and Figure 4). The AUC (0.792) confirmed that the WtHR has an acceptable discriminating ability, at almost 80% (79.2%), for detecting central obesity (Table 4 and Figure 3a).

Among White females, the mean age was 40.4 ± 11.3 years, the mean DXA-derived VAT area was 95.0 ± 40.1 cm^2^, and the mean WtHR was 0.57 ± 0.04. The majority (97.3%) were classified as having central obesity based on the arbitrary cut-off point (≥0.5) (Table 3). The ROC analysis for White females revealed that the most appropriate cut-off for detecting central obesity based on the DXA-derived VAT area tertiles was 0.58 (Table 4). This cut-off point achieved good sensitivity (72.6%) and specificity (75.1%). In relative terms, the proportion correctly diagnosed by the new cut-off point increased by 18.2%, attaining almost 60% (59.1%) (Table 5 and Figure 4). The AUC (0.824) showed that the WtHR has an excellent discriminating ability, at over 80% (82.4%), for identifying central obesity (Table 4 and Figure 3a).

For Black males, the mean age was 40.2 ± 11.5 years, the mean DXA-derived VAT area was 82.4 ± 33.2 cm^2^, and the mean WtHR was 0.53 ± 0.04. More than three quarters (78.5%) were categorized as having central obesity based on the arbitrary cut-off point (≥0.5) (Table 2). The ROC analysis for Black males showed that the most appropriate cut-off for identifying central obesity based on the DXA-derived VAT area tertiles was 0.55 (Table 4). This cut-off point achieved good sensitivity (73.5%) and specificity (77.7%). In comparative terms, the proportion correctly diagnosed by the new cut-off point improved by 17.4%, reaching almost 60% (59.6%) (Table 5 and Figure 4). The AUC (0.855) demonstrated that the WtHR has an excellent discriminating ability, at almost 86% (85.5%), for detecting central obesity (Table 4 and Figure 3b).

Among Black females, the mean age was 40.9 ± 11.6 years, the mean DXA-derived VAT area was 75.5 ± 31.1 cm^2^, and the mean WtHR was 0.56 ± 0.04. The majority (95.5%) were classified as having central obesity based on the arbitrary cut-off point (≥0.5) (Table 3). The ROC analysis for Black females confirmed that the most appropriate cut-off for detecting central obesity based on the DXA-derived VAT area tertiles was 0.57 (Table 4). This cut-off point achieved good sensitivity (71.8%) and specificity (68.9%). The proportion correctly diagnosed by the new cut-off point rose by 18.4%, attaining 53% (Table 5 and Figure 4). The AUC (0.763) confirmed that the WtHR has an acceptable discriminating ability, at almost 80% (76.3%), for identifying central obesity (Table 4 and Figure 3b).

For Asian males, the mean age was 41.5 ± 10.6 years, the mean DXA-derived VAT area was 111.3 ± 35.6 cm^2^, and the mean WtHR was 0.56 ± 0.03. The majority (96.5%) were categorized as having central obesity based on the arbitrary cut-off point (≥0.5) (Table 2). The ROC analysis for Asian males showed that the most appropriate cut-off for determining central obesity based on the DXA-derived VAT area tertiles was 0.56 (Table 4). This cut-off point achieved good sensitivity (72.6%) and specificity (65.5%). In comparative terms, the proportion correctly diagnosed by the new cut-off point increased by 16.8%, reaching 50% (51.0%) (Table 5 and Figure 4). The AUC (0.769) indicated that the WtHR has an acceptable discriminating ability, at over 75% (76.9%), for detecting central obesity (Table 4 and Figure 3c).

Among Asian females, the mean age was 42.9 ± 10.6 years, the mean DXA-derived VAT was 102.3 ± 32.8 cm^2^, and the mean WtHR was 0.58 ± 0.04. The majority (99.0%) were classified as having central obesity based on the arbitrary cut-off point (≥0.5) (Table 3). The ROC analysis for Asian females confirmed that the most appropriate cut-off for identifying central obesity based on DXA-derived VAT area tertiles was 0.59 (Table 4). This cut-off point achieved good sensitivity (73.9%) and specificity (73.6%). Comparatively, the proportion correctly diagnosed by the new cut-off point improved by 26.9%, attaining 61% (60.8%) (Table 5 and Figure 4). The AUC (0.834) demonstrated that the WtHR has an excellent discriminating ability, at over 80% (83.4%), for determining central obesity (Table 4 and Figure 3c).

For Hispanic males, the mean age was 40.2 ± 11.1 years, the mean DXA-derived VAT area was 110.7 ± 36.1 cm^2^, and the mean WtHR was 0.57 ± 0.03. The majority (96.8%) were categorized as having central obesity based on the arbitrary cut-off point (≥0.5) (Table 2). The ROC analysis for Hispanic males showed that the most appropriate cut-off for identifying central obesity based on the DXA-derived VAT area tertiles was 0.57 (Table 4). This cut-off point achieved good sensitivity (73.0%) and specificity (70.5%). In comparative terms, the proportion correctly diagnosed by the new cut-off point was improved by 19.7%, reaching almost 55% (54.3%) (Table 5 and Figure 4). The AUC (0.779) confirmed that the WtHR has an acceptable discriminating ability, at almost 80% (77.9%), for detecting central obesity (Table 5 and Figure 3d).

Among Hispanic females, the mean age was 40.0 ± 11.0 years, the mean DXA-derived VAT area was 100.8± 34.5 cm^2^, and the mean WtHR was 0.59 ± 0.04. The majority (98.9%) were classified as having central obesity based on the arbitrary cut-off point (≥0.5) (Table 3). The ROC analysis for Hispanic females showed that the most appropriate cut-off for determining central obesity based on the DXA-derived VAT area tertiles was 0.59 (Table 4). This cut-off point achieved good sensitivity (74.3%) and specificity (70.3%). Comparatively, the proportion correctly diagnosed by the new cut-off point was improved by 20.6%, attaining almost 55% (54.2%) (Table 5). The AUC (0.771) indicated that the waist-to-height ratio has an acceptable discriminating ability, at almost 80% (77.1%), for detecting central obesity (Table 5 and Figure 3d).

## 4. Discussion

The current study aimed to determine WtHR cut-off points that better discriminate central obesity in adults with overweight according to the WHO BMI classification of both sexes and different ethnicities, using data from the NHANES population in the US.

### 4.1. Findings and Comparison with the Previous Literature

In the current population-based study, our main finding was the identification of new WtHR cut-off points that better discriminate central adiposity in participants with overweight according to the WHO BMI classification (BMI = 25.0–29.9 kg/m^2^), based on data covering four different ethnicities from the NHANES in the US. These new WtHR cut-off points identified in the NHANES population across all different ethnicities and sexes were ≥0.55, slightly higher than the one (i.e., WtHR ≥ 0.5) recently suggested by some scientific organizations (i.e., the EASO framework) [31]. Notably, all the new cut-offs proposed for each ethnicity and sex, when compared to ≥ 0.5, appear to improve the performance of the WtHR in determining central adiposity. In other words, the new cut-offs in all ethnicities and sexes (≥0.55) generally increase the correct identification rate for cases and reduce the false positives [39].

Although several works are available on the identification of WtHR cut-offs for screening cardiometabolic diseases in adults [40,41,42], to date, few analyses have tried to establish WtHR cut-offs as indicators of adiposity, especially central obesity [29]. Remarkably, the cross-sectional investigation conducted by Roriz et al. among a sample of 191 adults reported WtHR cut-off points ranging from 0.54 to 0.59 for predicting high visceral adiposity (a VAT area of ≥130 cm^2^ determined by CT) in men and women aged 20–59 years and ≥60 years, respectively [43]. These cut-offs are very similar to those reported in our paper, which, despite the small sample size, relied on a gold standard assessment of VAT (i.e., the CT scan), as well as defined cut-offs of the latter that indicated central obesity [43].

### 4.2. Clinical Implications

This finding has several implications. Firstly, while the assessment of VAT is usually costly and requires specialized equipment and, in some cases, specific skills, we strongly encourage the use of the WtHR, which would allow for easy-to-obtain adiposity measures. Secondly, policy-makers, at least those in the US, are invited to take these results as preliminary evidence of new cut-off points for identifying central adiposity in this specific population (i.e., the overweight NHANES population). Finally, awareness should be raised among all healthcare professionals dealing with obesity in regard to recognizing these new cut-off points when screening for central adiposity and sharing or discussing this new information with their patients.

### 4.3. Strengths and Limitations

This study has certain strengths. Primarily, it considered a large sample of participants with an overweight status according to WHO BMI categorization, including four different ethnicities and both sexes, and relied on a reliable and validated parameter to determine central adiposity, in the form of DXA-derived VAT. It is one of the very few works to derive tentative cut-off points for the WtHR for the prediction of high DXA-derived VAT. However, at the same time, our paper also has certain limitations. Firstly, as mentioned above, there is no consensus regarding the definition of central obesity based on DXA-derived VAT. Defining the latter as the highest VAT tertile within each ethnicity may therefore be criticized. Secondly, the fact that we studied a specific population (i.e., young and middle-aged adults, people living in the US, etc.) means that our findings cannot be generalized to others (i.e., older adults, people outside the US, etc.), and our investigation thus lacks external validity in different settings [44]. Thirdly, DXA software (Hologic APEX 4.0) was employed to estimate the VAT area, and although it is an acceptable and validated technique [45], it is not considered a gold standard measure for this purpose when compared with other methods [46]. Finally, this investigation adopted a cross-sectional design [47] and was therefore unable to detect DXA-derived VAT trends or changes [48], which usually requires longitudinal assessment [49].

### 4.4. New Directions for Future Research

Some new directions for future research derived from our findings still need to be explored. Firstly, additional investigations should replicate our findings to confirm these WtHR cut-off points in the US, perhaps in a purely real-world clinical setting (i.e., primary care, clinical specialized units, etc.). Moreover, subsequent studies are also needed to determine the WtHR cut-off points in individuals with a normal weight (BMI 18.5–24.9 kg/m^2^) or obesity (BMI ≥ 30 kg/m^2^) based on WHO BMI categorization. Finally, other works should extend our analysis to different populations worldwide.

## 5. Conclusions

Central visceral obesity is clinically associated with severe comorbidities, and its early identification, especially by means of surrogate measures such as the WtHR, is thus crucial for managing the progression of the latter. In this study, we provide evidence that the optimal WtHR cut-off point (i.e., in all cases ≥ 0.55) corresponds to central obesity in adults of a both-sex population composed of different ethnicities. We therefore recommend the use of these new WtHR cut-off points when screening individuals for central obesity while taking into account their ethnicity and sex.

## Figures and Tables

**Table 1:** Anthropometric characteristics and body composition of the total study sample by ethnicity (*n* = 3566).

		Ethnicity			
	Total (*n* = 3566)	White (*n* = 1285)	Black (*n* = 722)	Asian (*n* = 536)	Hispanic (*n* = 1023)
Age	40.6 (11.1)	40.1 (11.2)	40.5 (11.5)	42.0 (10.6)	40.1 (11.1)
Sex					
Males	2077 (58.2)	760 (59.1)	410 (56.8)	342 (63.8)	565 (55.2)
Females	1489 (41.8)	525 (40.9)	312 (43.2)	194 (36.2)	458 (44.8)
BMI (kg/m^2^)	27.4 (1.4)	27.4 (1.4)	27.4 (1.4)	27.2 (1.4)	27.6 (1.4)
Weight (kg)	78.2 (10.1)	81.3 (9.8)	80.2 (9.3)	74.6 (9.3)	74.9 (9.6)
Height (cm)	166.7 (10.1	168.9 (9.9)	168.9 (9.6)	163.2 (9.4)	163.1 (9.5)
WC (cm)	94.9 (6.8)	96.7 (6.9)	93.3 (7.0)	93.7 (6.2)	94.4 (6.4)
WtHR	0.56 (0.04)	0.56 (0.04)	0.55 (0.04)	0.57 (0.04)	0.58 (0.04)
<0.5	200 (5.6)	61 (4.7)	102 (14.1)	14 (2.6)	23 (2.2)
≥0.5	3366 (94.4)	1224 (95.3)	620 (85.9)	522 (97.4)	1000 (97.8)
DXA-derived VAT area (cm^2^)	100.5 (38.8)	104.6 (41.9)	79.4 (32.4)	108.0 (34.9)	106.2 (35.7)

BMI = body mass index; WC = waist circumference; WtHR = waist-to-height ratio.

**Table 2:** Anthropometric characteristics and body composition among males by ethnicity (*n* = 2077).

		Ethnicity			
	Total (*n* = 2077)	White (*n* = 760)	Black (*n* = 410)	Asian (*n* = 342)	Hispanic (*n* = 565)
Age	40.6 (11.1)	40.8 (11.1)	40.2 (11.5)	41.5 (10.6)	40.2 (11.1)
BMI (kg/m^2^)	27.4 (1.4)	27.4 (1.4)	27.3 (1.4)	27.1 (1.3)	27.7 (1.4)
Weight (kg)	83.4 (8.4)	86.5 (8.0)	85.3 (7.7)	79.2 (7.5)	80.5 (7.9)
Height (cm)	174.2 (7.7)	177.4 (6.8)	176.6 (6.8)	170.7 (7.1)	170.4 (7.3)
WC (cm)	96.6 (6.6)	98.7 (6.4)	94.4 (7.5)	95.2 (5.8)	96.5 (6.0)
WtHR	0.56 (0.04)	0.56 (0.04)	0.53 (0.04)	0.56 (0.03)	0.57 (0.03)
<0.5	165 (7.9)	47 (6.2)	88 (21.5)	12 (3.5)	18 (3.2)
≥0.5	1912 (92.1)	713 (93.8)	322 (78.5)	330 (96.5)	547 (96.8)
DXA-derived VAT area (cm^2^)	105.4 (39.3)	111.2 (41.8)	82.4 (33.2)	111.3 (35.6)	110.7 (36.1)

BMI = body mass index; WC = waist circumference; WtHR = waist-to-height ratio.

**Table 3:** Anthropometric characteristics and body composition among females by ethnicity (*n* = 1489).

Females		Ethnicity			
	Total (*n* = 1489)	White (*n* = 525)	Black (*n* = 312)	Asian (*n* = 194)	Hispanic (*n* = 458)
Age	40.7 (11.2)	40.4 (11.3)	40.9 (11.6)	42.9 (10.6)	40.0 (11.0)
BMI (kg/m^2^)	27.4 (1.4)	27.3 (1.4)	27.6 (1.4)	27.2 (1.4)	27.6 (1.4)
Weight (kg)	71.0 (7.3)	73.7 (6.9)	73.6 (6.6)	66.6 (6.1)	68.0 (6.5)
Height (cm)	160.7 (7.3)	164.1 (6.4)	163.3 (6.5)	156.4 (5.9)	157.0 (6.5)
WC (cm)	92.5 (6.4)	93.8 (6.7)	91.9 (6.2)	91.1 (6.1)	91.9 (6.1)
WtHR	0.58 (0.04)	0.57 (0.04)	0.56 (0.04)	0.58 (0.04)	0.59 (0.04)
<0.5	35 (2.4)	14 (2.7)	14 (4.5)	2 (1.0)	5 (1.1)
≥0.5	1454 (97.6)	511 (97.3)	298 (95.5)	192 (99.0)	453 (98.9)
DXA-derived VAT area (cm^2^)	93.6 (37.0)	95.0 (40.1)	75.5 (31.1)	102.3 (32.8)	100.8 (34.5)

BMI = body mass index; WC = waist circumference; WtHR = waist-to-height ratio.

**Table 4:** Diagnostic performance of the new WtHR cut-off points for central obesity across sex and ethnicity groups (*n* = 3566).

	*n*	AUC	95%CI	*p* Value	Cut-Off	Sensitivity	Specificity
Males							
White	760	0.7920	0.7563–0.8230	<0.0001	0.57	0.7183	0.7461
Black	410	0.8550	0.8145–0.8872	<0.0001	0.55	0.7353	0.7774
Asian	342	0.7695	0.7125–0.8165	<0.0001	0.56	0.7257	0.6550
Hispanic	565	0.7785	0.7356–0.8151	<0.0001	0.57	0.7302	0.7048
Females							
White	525	0.8241	0.7850–0.8566	<0.0001	0.58	0.7257	0.7514
Black	312	0.7632	0.7046–0.8114	<0.0001	0.57	0.7184	0.6890
Asian	194	0.8342	0.7660–0.8839	<0.0001	0.59	0.7385	0.7364
Hispanic	458	0.7710	0.7228–0.8118	<0.0001	0.59	0.7434	0.702

**Table 5:** Proportion correctly diagnosed by ≥0.5 cut-off point and new cut-off points (*n* = 3566).

	Total Sample			WtHR ≥ 0.5			New Cut-Off Point ≥ C^§^			Δ % Detected
		DXA-Derived VAT (cm^2^)		Total Classified ≥ 0.5	Proportion Correctly Diagnosed (TP)	Proportion Incorrectly Diagnosed (FP)	Total Classified ≥ C^§^	Proportion Correctly Diagnosed (TP)	Proportion Incorrectly Diagnosed (FP)	
		3rd Tertile	1st and 2nd Tertiles							
	*n* (%)			*n* (%)			*n* (%)			
Males										
White	760 (100)	252 (33.2)	508 (66.8)	713 (100)	252 (35.3)	461 (64.7)	273 (100)	163 (59.7)	110 (40.3)	+24.4
Black	410 (100)	136 (33.2)	274 (66.8)	322 (100)	186 (57.8)	136 (42.2)	183 (100)	109 (59.6)	74 (27.0)	+17.4
Asian	342 (100)	113 (33.0)	229 (66.9)	330 (100)	113 (34.2)	217 (65.8)	155 (100)	79 (51.0)	76 (49.0)	+16.8
Hispanic	565 (100)	189 (33.5)	376 (66.5)	547 (100)	189 (34.6)	358 (65.4)	254 (100)	138 (54.3)	116 (45.7)	+19.7
Females										
White	525 (100)	175 (33.3)	350 (66.7)	511 (100)	175 (34.2)	336 (65.8)	215 (100)	127 (59.1)	88 (40.9)	+18.2
Black	312 (100)	103 (33.0)	195 (62.5)	298 (100)	103 (34.6)	195 (65.5)	132 (100)	70 (53.0)	62 (47.0)	+18.4
Asian	194 (100)	65 (33.5)	129 (66.5)	192 (100)	65 (33.9)	127 (66.1)	79 (100)	48 (60.8)	31 (39.2)	+26.9
Hispanic	458 (100)	152 (33.2)	306 (66.8)	453 (100)	152 (33.6)	301 (98.4)	212 (100)	115 (54.2)	97 (45.8)	+20.6

C^§^ New sex and ethnicity specific WtHR cut-off point; TP = true positive; FP = false positive.

## Data Availability

The original data presented in the study are openly available on the following site: https://wwwn.cdc.gov/nchs/nhanes/Default.aspx (accessed on 10 September 2024).
